# Crimean-Congo Hemorrhagic Fever Asia-2 Genotype, Pakistan

**DOI:** 10.3201/eid1906.120771

**Published:** 2013-06

**Authors:** Muhammad Masroor Alam, Adnan Khurshid, Salmaan Sharif, Shahzad Shaukat, Rana Muhammad Suleman, Mehar Angez, Syed Sohail Zahoor Zaidi

**Affiliations:** National Institute of Health, Chak Shahzad, Islamabad, Pakistan

**Keywords:** Crimean-Congo Hemorrhagic Fever, Bunyaviridae, Nairovirus, viruses, Pakistan, Baluchistan, CCHF, CCHFV, Asia-2 genotype, molecular epidemiology, tickborne, zoonotic, zoonoses, migratory birds

**To the Editor:** Crimean-Congo hemorrhagic fever (CCHF) is a tickborne zoonotic disease caused by a member of the virus family *Bunyaviridae*, genus *Nairovirus*. This virus (CCHFV) has caused illness throughout Asia, Europe, Africa, and the Middle East (*1*). CCHFVs are clustered among 7 genotypes (Asia-1, Asia-2, Euro-1, Euro-2, Africa-1, Africa-2, and Africa-3) on the basis of genetic variation in the small segment (*2*). These genotypes are well conserved among their regions of origin; however, >1 genotype is prevalent in many countries (*2*). In Pakistan, the first CCHF case was reported in 1976; multiple sporadic cases and outbreaks have occurred in subsequent years (*3*). 

To determine which genotypes were present in Pakistan, we performed molecular analysis of archived serum samples collected during 2008 in Fatima Jinnah General and Chest Hospital, Quetta, Baluchistan, in southwestern of Pakistan. Because of limited diagnostic facilities for CCHFV in this country, samples collected during 1976–2002 were occasionally sent to laboratories in countries such as South Africa and the United States, where genetic analysis showed that all viruses tested from that location belonged to the Asia-1 genotype (*4*). Data beyond this period are not available; however, because of improved molecular diagnostic facilities at the Department of Virology, National Institute of Health, Pakistan, blood samples collected from patients with suspected cases attending in-country hospitals are now examined by the institute for confirmation. Our findings substantiate the presence of Asia-1 and Asia-2 genotypes in Baluchistan.

Thirteen IgM-positive samples collected during 2008 and stored at –70°C were available for study. The samples were processed for amplification of 260 bp of the small segment by using reverse transcription PCR with a previously described protocol (*5*). The mean age of patients with serology-confirmed CCHF was 31.3 (range 18–40) years; male-to-female IgM positivity ratio was 1:2. Common symptoms were fever, headache, and nosebleeds. Platelet counts ranged from 16,000 to 43,000/μL of blood. 

Of the 13 samples, viral RNA was detected in 2 (CCHF-65–2008PAK and CCHF-43–2008PAK); the amplicons were subjected to bidirectional sequencing by using the BigDye Terminator v3.1 cycle sequencing kit (Applied BioSystems, Foster City, CA, USA). Sequences were analyzed with Sequencher (GeneCodes Corp., Ann Arbor, MI, USA) and MEGA v4.0 (http://megasoftware.net/). The 2 viruses were phylogenetically clustered into Asia-1 and Asia-2 genotypes, with 7% nucleotide divergence, although both samples were collected during September–October, 2008. 

The closest nucleotide identity (99%–100%) for CCHF-65–2008PAK was found with the previously reported Asia-1 strains from Pakistan, Afghanistan, and Iran; CCHF-43–2008PAK had 96%–97% similarity to viruses from Dubai and Tajikistan (Figure). The sequences reported from United Arab Emirates, Pakistan, Afghanistan, Iran, and Iraq belong to the Asia-1 genotype; the Asia-2 genotype sequences were mostly from China and Central Asian countries such as Uzbekistan, Tajikistan, and Kazakhstan (*6*). All viruses detected intermittently in Pakistan during 1976–2002 were of the Asia-1 genotype (*4*). However, the analysis of the 2 samples reported here enhances our knowledge of CCHFV genetic diversity in Pakistan. 

**Figure:**
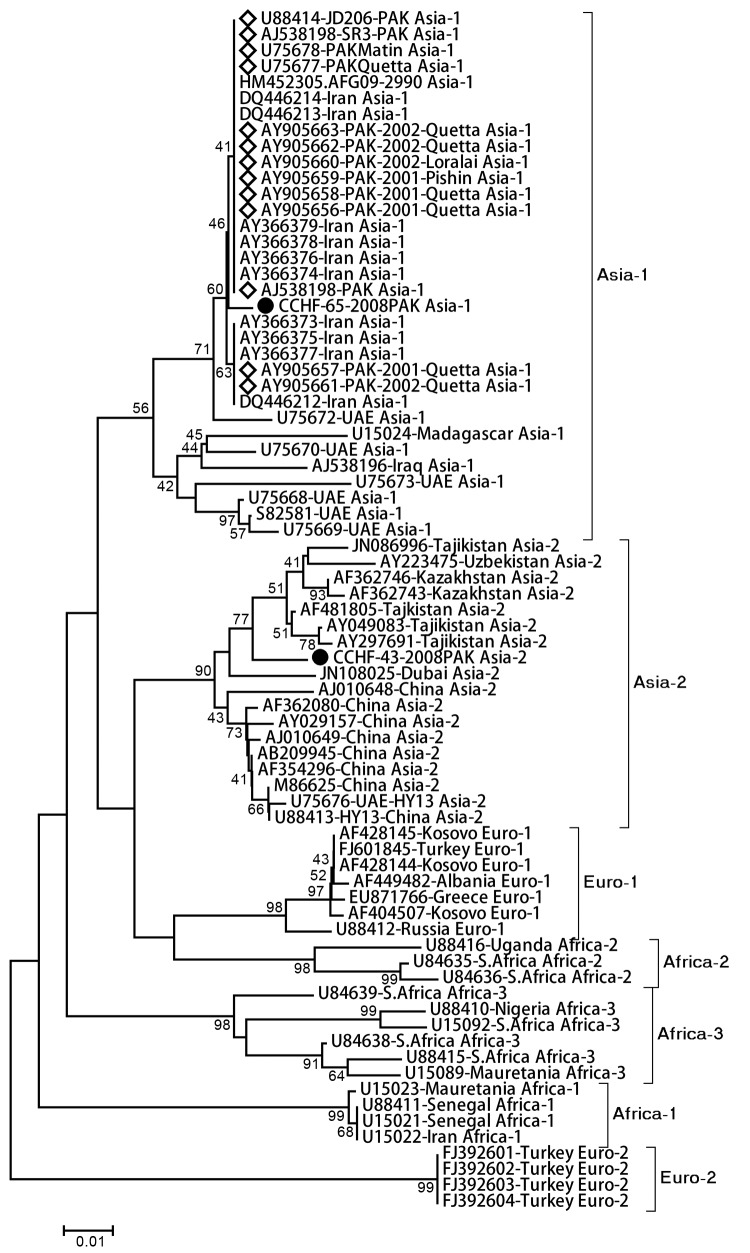
Phylogenetic analysis of partial small gene fragment (220 bp) obtained from Crimean-Congo hemorrhagic fever virus strains analyzed in this study (black circles). Reference strains belong to different genogroups as retrieved from GenBank. Diamonds indicate virus sequences previously reported from Pakistan. Evolutionary tree and distances (scale bar indicates number of base substitutions per site) were generated with the maximum composite likelihood method with Kimura-2 parameter distances by using MEGA 4.0 (http://megasoftware.net/). Numbers next to branches indicate the percentage of replicate trees in which the associated taxa clustered together in the bootstrap test (1,000 replicates). The GenBank accession numbers, country, year of sample collection, and respective genotype information have been provided where available.

The closest phylogenetic positioning of CCHF-43–2008PAK with Asia-2 strain Dubai-616 (GenBank accession no. JN108025) indicates that the probable route of CCHFV transmission was through animal trade between the United Arab Emirates and Pakistan. This finding supports the proposition that animals imported from Pakistan were the probable source of a 1979 outbreak in the United Arab Emirates (*7*). However, we cannot determine the direct source of the Asia-2 genotype in Pakistan, nor confirm the transmission link between the 2 countries. We attribute this to a lack of consistent, contemporary viral genetic information of CCHFV strains in Pakistan and the United Arab Emirates. This lack of data necessitates intensive surveillance and epidemiologic investigations in animal and human populations because geographic factors alone do not provide comprehensive information about the diversity of CCHFV strains circulating in Asia (*6*).

The presence of geographically distant, but genetically similar, strains suggests that the viruses are dispersed either through animal trade or migratory birds (*8*). No clear evidence of CCHFV infection in migratory birds has been found, but they may play a major role in translocation of infected ticks to distant areas (*9*). Birds are known to be parasitized by these vectors of CCHFV in eastern Europe and Asia and disseminate the virus by transporting infected immature ticks between continents (*4*). It is therefore highly advisable to develop an active surveillance system with appropriate laboratory facilities to conduct the seroepidemiologic surveys and screening of household animals and vectors for CCHFV to rule out potential risks. 

Our study was limited by a low number of samples, resulting in availability of only a short fragment of the small gene for analysis. However, similar partial small gene sequencing has been used in previous studies (*2*) and has supported the classification of CCHFV strains correctly into 7 genotypes.

In conclusion, because tick control is not feasible, surveillance activities and laboratory facilities should be improved. Health care workers should also be aware of proper patient management and standard prophylactic and preventive measures, particularly in areas where CCHFV is endemic, such as Baluchistan, where many deaths associated with nosocomial transmission have been reported (*10*).
